# Clinical outcome and technical complications of bimaxillary full-arch implant-supported metal-resin fixed dental prostheses with or without ceramic molars: 5-year results

**DOI:** 10.1007/s00784-025-06409-y

**Published:** 2025-06-05

**Authors:** Stefan Krennmair, Michael Weinländer, Lukas Postl, Michael Malek, Thomas Forstner, Helfried Hulla, Gerald Krennmair

**Affiliations:** 1 Department of Oral and Maxillofacial Surgery, Keplerklinikum Linz, Johannes Kepler University (JKU) Linz, Linz, Austria; 2 NumBiolab Research Associate, Ludwig-Maximilian University (LMU), Munich, Germany; 3 Dental School, Medical University Graz, Graz, Austria; 4 Head of Department of Oral and Maxillofacial Surgery, Keplerklinikum Linz, Johannes Kepler University (JKU) Linz, Linz, Austria; 5 Department of Applied Systems and Statistics, Johannes Kepler University Linz (JKU), Linz, Austria; 6 Head of Department of Prosthodontics, Dental School, Sigmund Freud Medical University of Vienna, Vienna, Austria

**Keywords:** Bimaxillary metal-resin prostheses, Dental implants, Technical complications, Denture modifications

## Abstract

**Purpose:**

To evaluate the prevalence of prosthetic-related technical complications (PRTC) for bimaxillary implant-supported fixed complete metal-resin prostheses (ISFP) with (Zrm-ISFP) or without (r-ISFP) occlusal support by incorporation of zirconium molars.

**Materials and methods:**

Two cohorts of patients with bimaxillary ISFP subdivided into test group (TG; Zrm-ISFP) and control group (CG, r-ISFP) providing a 5-year follow-op program were retrospectively analyzed. For both groups the prevalence of PRTC was assessed and compared including evaluation of their occurrence in different time periods (-1 year, > 1–3-years; > 3–5-years post-loading). Additionally, implant/prosthesis survival-rates, peri-implant-marginal bone loss (MBL) and implant-related-technical complications were evaluated and compared.

**Results:**

For the 29 patients with bimaxillary ISFP subdivided into 14 TG [Zrm-ISFP] and 15 CG [r-ISFP]) and followed-up for 5 years no implant and denture loss was seen (survival 100%). However, the prevalence of PRTC such as acrylic-tooth fracture (p < 0.001) and acrylic-tooth repair (p = 0.015) differed significantly between TG (Zrm-ISFP) and CG (r-ISFP). For the CG, an evidently higher time-related ongoing increase (-1 yr: n = 9; > 1-3yrs: n = 23; > 3-5yrs: n = 34) of the prevalence of PRTC was noted compared to TG ( -1 yr: n = 2; > 1-3yrs: n = 3; > 3-5yrs: n = 7). In contrast, PRTC such as denture rebasing/reduction, denture cleaning, screw hole repair and implant-related technical complications and peri-implant MBL did not differ between TG and CG.

**Conclusion/clinical relevance:**

The modified metal-resin ISFPs incorporating zirconium molars/quadrants used for bimaxillary ISFP reduce the prevalence of PRTC and combine beneficial effects such as cost effectiveness and reparability of metal-resin and occlusal stability and reduced wear of complete zirconium prostheses.

## Introduction

Implant-supported fixed full-arch prostheses (ISFP) represent a viable treatment option for rehabilitation of edentulism [1–5]. However, in addition to investigations of the implant and prosthesis survival rate, evaluations of the biological and technical complications and the associated post-rehabilitation support have gained in importance [6–10].

Numerous studies, systematic reviews and meta-analyses have shown that the most common fabrication modality for ISFP involves metal resin [3, 5, 11–14]. Long-term records of metal-resin ISFP showed high implant-prosthodontic success rates, high patient satisfaction, simplicity in use, reduced fabrication costs, and ease of reparability[3, 5, 13, 14]. However, high complication rates for metal-resin ISFP reported as denture teeth debonding and veneered acrylic teeth fracture are time-consuming for both patients and clinicians and have led to the consideration of potential alternatives [3, 5, 14–16]. As alternatives to metal-resin reconstructions ISFP may also be fabricated from modern synthetic materials, fully ceramic material (monolithic or veneered) or metal-ceramic material [17–21]. These materials vary in their production and fabrication, their purchase and maintenance costs and also their maintenance efforts and expenses [22]. While comparative investigations of metal-acrylic and full-ceramic prostheses showed high implant/prosthesis and patient success rates, the studies revealed significant advantages in favor of full ceramic materials with respect to the aesthetic appearance achieved, the occlusal stability and resistance and a reduced plaque accumulation [22, 23]

It is a well-known fact that the acrylic teeth of a metal-resin ISFP will be subject to occlusal wear and may thus be associated with a reduction of occlusal stability and consequently with a loss of occlusal surface [14–16]. These prosthesis-related technical complications may be the result of the synthetic prosthesis material used and may also be attributed to the type and the material used for the restoration of the opposite jaw [24–26]. The fact that the type and form of the opposing jaw will have an impact on the complication rate had already been described by the study of Davis et al.[25] in 2003, in which they were able to show that complications of an ISFP opposed by fixed prostheses were much more frequent and more pronounced than when opposed by natural teeth or complete dentures [25.26]. In this context, Malo et al. [27] as well as Papaspyriakos et al.[28] were also able to show that bimaxillary fixed implant-prosthetic restorations – regardless of the material used – were associated with a significantly higher rate of prosthesis-related technical complications than monomaxillary restorations [27–29].

Because of the high number of technical complications seen with bimaxillary metal-resin ISFP alternative modifications have been developed for this kind of prosthesis [30, 31]. In this respect a specific modification of the bimaxillary metal-resin ISFP has been described in separate studies by AlHelal et al.[30] and AlBader et al.[31] incorporating a metal-occlusal surface of the molars into the metal-resin prosthesis. With this incorporated occlusal surface in the molar region abrasion-resistant occlusal stability will be achieved consequently providing for a reduction of the prosthetic complication rate [11, 15]. In addition to the attempts of using and integrating metal inlays/onlays in the occlusal prosthetic design, the incorporation of singular zirconium crowns in molar positions may also be considered as alternative for maintaining occlusal stability [30, 31]. However, although it is a well-known fact that maintenance of occlusion will especially contribute to the reduction of technical complications, the incorporation of zirconium molar crowns into the metal-resin IFSP for the purpose of occlusal stabilization has not yet been described in any study or investigation [14, 15, 30, 31].

The primary objective of the present study was to evaluate the prosthesis-related technical complication rate of bimaxillary metal-resin ISFP with zirconium molar (Zrm-ISFP) over a follow-up period of 5 years and to compare it with that of IFSP without incorporated zirconium crown (r-ISFP). As a secondary objective, the implant/prosthesis survival rates and the implant-related technical complications in the 1-, 3- and 5-year period of follow-up time were evaluated and compared between the two groups. Initially, it was hypothesized that no difference would be observed between Zr-ISFP and r-IFSP for the implant-related survival rate and the prosthesis-related technical complication rate.

## Material and methods

### Study design/patients

The present study was designed as a single-center retrospective follow-up evaluation and included patients with bimaxillary implant-supported fixed full arch prostheses (ISFP) representing a 5-year postinsertion observation period. In relation to the different denture design and time periods of fabrication the retrospectively evaluated population could be subdivided into two cohorts: 1) the test-group (TG) and −2) the control-group (CG).

The test-group (TG) consisted of 18 patients with bimaxillary ISFP made between January 2017 and September 2019 in whom the metal-resin denture design was additionally provided with the incorporation of a zirconium molar/quadrant (TG, Zrm-ISFP). In relation to the number of patients of the test group retrospectively recruited a valid group of edentulous patients rehabilitated with bimaxillary metal-resin ISFP (without ceramic-molars) treated before 2017 serving as control group (control group; CG, r-ISFP) was additionally enrolled from patient charts. Patients of both groups had to fulfill the main inclusion criteria representing a continuous 5-years observation period. Data of the epidemiologic, implant-surgical as well implant-prosthodontic characteristics of the included test group (TG) and the matchable control group (CG) allowing for group comparisons are presented in Table 1.

**Table 1:** Patient characteristics and demographic data of subjects with bimaxillary implant supported fixed metal-resin prostheses either with (TG:Zrm-ISFP) or without zirconium molars (CG:r-ISFP)

	Zrm—ISFP	r – ISFP
Pat:	n = 14	n = 15
Age (yrs):	58.6 ± 8.3	56.2 ± 10.8
Sex (f/m)	8/6 (57.1%/42.8%)	7/8 (46.7%/%/53.3%)
Smoker:	3/14 (21.4%)	2/15 (13.3%)
Diabetes I:	1/14 (7.1%)	0
Rheuma:	1/14 (7.1%)	0
Implant-prosthodontic:		
Mandible: “All-on 4”:	14	15
Implants mandible:	56	60
Maxilla: “All-on 4/5/6”:	4/7/3	4/8/3
Implants maxilla:	69	74

Each patient evaluated was given a detailed description of the procedures and signed an informed consent prior to enrollment. The investigation was conducted according to the principles embodied in the Helsinki Declaration for biomedical research involving human subjects. The study protocol was approved by the local ethics committee (EK-SFU: 1198–2025), was self-funded by the authors and their institutions and was reported according the STROBE guidelines for cohort studies (http://www.strobe-statement.org).

### Inclusion criteria/implant placement

Table 2 presents the initial inclusion and exclusion criteria for patients scheduled for implant placement and subsequent implant prosthodontic treatment. Inclusion criteria as well as the surgical procedure followed were identical for all patients admitted either for the test (TG, Zrm-ISFP) or for the control (CG, r-ISFP) group.

**Table 2:** Inclusion and exclusion criteria for bimaxillary ISFP

Inclusion criteria	Exclusion criteria:
Edentulism - Healed sites or residual dentition wish & need fixed implant prostheses No sinus pathology Implants at least: > 11 mm in length > 3.8 mm in diameter Age: > 18 yrs oral hygiene recall	untreated periodontitis Sinus pathology Uncontrolled systemic disease: rheumatic disorders/bisphosphonate therapy uncontrolled diabetes mellitus (HbA1 C > 7.5% alcoholism/drug abuse severe bruxism (CMD disorders) lack of compliance History of radiation therapy head/neck physical handicap

For all patients included initially before implant placement, impressions were done for preparing models and documents producing provisional prostheses. One day before surgery patients received antibiotic prophylaxis (amoxicillin 2 × 1 g) being continued for 6 days postoperatively. Surgical procedures were performed under local anesthesia (articaine-chlorohydrate and epinephrine 1:100000).

In cases of immediate implant placement, when residual teeth were present the teeth were carefully extracted and the alveolar sockets were carefully and thoroughly debrided and using a periodontal probe the bony walls of the fresh extraction sites were examined for integrity. As described in previous studies the implant platform was positioned slightly subcrestally in healed sites, while in extraction sites the platform was placed slightly deeper subcrestally (1–2 mm) to minimize the vertical gap [32]. Residual empty alveolar sockets were filled with a mixture of autogenous bone chips (drill harvesting) and bovine bone mineral (Bio-Oss®, Geistlich, Wollhusen) [32].

In all patients, 4 interforaminal implants were placed in the mandible according to the “All-on-4” protocol with 2 anterior implants placed in an axial position (position of the lateral incisors) and the 2 posterior implants as close as possible to the mental foramen in a distally tilted direction (position at 1 st or 2nd premolar) [33, 34]. Between the axial anterior and tilted posterior implants an optimal anterior–posterior spread was intended for creating a sufficient supporting zone for full-arch prosthesis stabilization and cantilevering [3, 5, 35, 36].

According to previously reported protocols implant placement in maxillary edentulism was performed either according to the anterior or posterior concept.^37^ For the anterior concept, 4–5 implants were placed in the pristine interantral maxillary region and for the posterior concept (6 implants) implant placement consisted of implants placed in the pristine canine region (2 implants) and augmented maxillary posterior regions (4 implants, posterior concept) [37, 38]. Maxillary posterior denture support was aimed at either with tilted posterior implants in native bone (anterior concept) including angulated abutments or with posterior axial implants with straight abutments in augmented maxillary sinus regions (posterior concept).^37^ For both groups evaluated, implants placed were of solid-screw tapered design with a medium-rough sand-blasted and acid-etched surface. Implant design varied in relation to placement time and could be divided into screw/root-line design (early phase, before 2017) and progressive design (later phase, placement after 2017). For all implants – either in mandible or maxilla—the insertion torque (IT) measurement was carried out at implant placement using a motor (Elcomed SA-310®; W&H, Bürmoos, Austria) and when an IT > 30 Ncm was obtained implants were considered for immediate loading with an interim prosthesis.

### Prosthetic procedure

In cases of immediate loading using interim acrylic prostheses following successful implant placement with a torque of > 30 Ncm selected abutments (straight or/and inclined, abutment height:0.5–1 mm) were screwed with a 20 Ncm force. After impression or scanning at abutment level temporary prostheses (up to 1 st molar/2nd premolar including a simple metal bar) were fabricated and inserted (20 Ncm) within 24 h. A soft diet was recommended for 4–6 weeks and oral hygiene instructions were given [37].

In cases with non-immediate implant loading or for immediate implant loading with temporary prostheses about 6 months after implant placement patients returned for fabrication of definitive maxillary and mandibular prostheses. For non-immediate loading submerged implants were uncovered and about 10 days later impression/scans were made at implant level. In cases with immediate implant loading (with interim prostheses) definitive impressions/scans were made at abutment level [37, 38].

After model production and abutment selection—if necessary for non-immediate loading – the procedure of prosthesis fabrication started. The models were produced and were mounted in centric relation at the proper vertical dimension of occlusion. Centric relation was recorded utilizing centric pin registration. After adjustment of the vertical relation, review of the wax-ups for phonetics and aesthetics, cobalt-chromium screw-retained frameworks were conventionally casted or digitally milled.

**Fig. 1:**
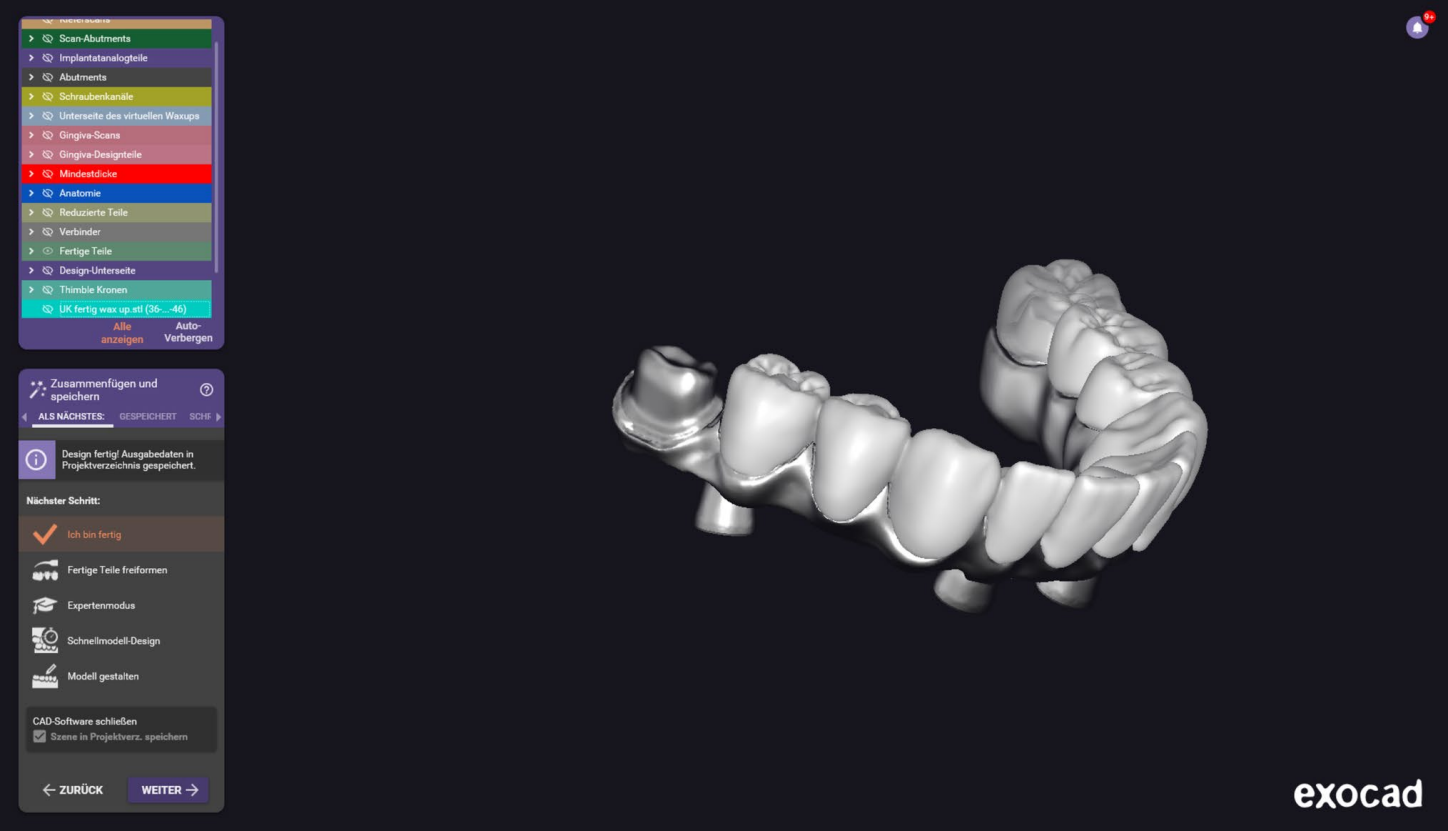
Milled (CAD/CAM) CO-Cr Framework for incorporation of zirkonium molars (Zrm-ISFP)

For the implant-supported fixed prostheses a metal framework was casted or CAD/CAM milled in Co-Cr metal alloy. For both the TG (Zrm-ISFP) and CG (r-ISFP) the metal framework provided retention devices for the acrylic teeth used. However, in the TG where the incorporation of zirconium molars was intended, a metal core for molar-crown fixation was additionally milled/casted within the Co-Cr framework (Fig. 1). Framework passivity was verified by a combination of methods such as the 1-screw test and radiographically with a panoramic radiograph, and/or visually. After framework try-in registration of the centric relation was repeated.

Framework production was followed by the final fabrication of the ISFP prostheses. The maxillary and mandibular prostheses were built either with 12–14 acrylic teeth (CG, r-ISFP) or with 10–12 acrylic teeth and one zirconium in 1 st-molar tooth position (TG,Zrm-ISFP). The number of molars (1 or 2) depended on the alveolar ridge length and the opposite jaw relations.

The fundamental prosthesis design such as framework characteristics, prosthesis base, retention modality for acrylic teeth including the lingual and basal metal surface was identical for Zrm-ISFP and r-ISFP [39, 40]. The occlusal scheme for both designs of the prosthesis was set to bilateral simultaneous posterior contacts in centric relation with canine guided occlusion in excursive movements. The access holes were filled with cotton pellets and a polyester teflon band and closed with composite resin (Tetric ceram—flow; Ivoclar Vivadent, Amherst, New York).

### Outcome measures

For all patients included primary outcome (prosthesis-related technical complications) and secondary outcome (implant-related complications) measurements were assessed at the scheduled 1-year, 3-year and 5-year follow-ups post functional denture loading as well as at unscheduled appointments. Primary and secondary outcome measurements were documented in the patient charts by a trained prosthodontic (S.K., M.W.,G.K.) and served for retrospective data analysis [32].

#### Primary outcome measures

The primary outcome measures included the assessment of the prevalence and kind of prosthesis-related technical complications as follows: framework fracture, denture fractures, denture teeth fracture, teeth abrasion, teeth crack, denture rebasing (performed with silicon impression; IMPRINT™, 3 M ESPE, Seefeld, Germany) as well as denture reduction. Prosthesis-related technical complications were either directly noticed at the 1-, 3- and 5-year clinical follow-up evaluation (such as abrasion and cracks), recorded at unscheduled visits at any time (i.e., teeth/denture fracture) or were noted at the annual hygienic program [6, 7]. The prevalence/kind of prosthesis-related technical complications recorded was summarized over the 5-year observation period and was also separated for occurrence in 3 different time periods (early period: up to 1 year post-loading; mid-term period: > 1–3 years post-loading; final observation period: > 3–5 years post-loading).

#### Secondary outcome measures

- Implant-related technical complications: included the evaluation of implant loosening, implant fracture, abutment fracture, abutment loosening, implant/abutment screw loosening [6, 10, 12].
- Peri-implant marginal bone level (MBL): panoramic radiographic evaluations (Orthophos XGPlus, Sidexis, Siemens, Sirona Dental System) including single periapical radiographs with the paralleling technique (long cone with standardized x-ray-holders) were done to assess the bone height level in relation to the implant shoulder (reference point). In relation to general crestal implant placement at baseline all implants placed were defined as 100% bone surrounded and the bone level at placement was set as zero level. Radiographs were taken: immediately postoperatively (baseline) after implant placement and at the follow-up evaluation and were compared to calculate the peri-implant MBL change and the peri-implant marginal bone loss as the result of the difference (Laurell & Lundgren, 2011)[40]. All radiographs were measured by an independent examiner (S.K.) who was trained in radiographic measuring and was blinded to the study [32]. Radiographic measuring was repeated within 2 weeks and the worst value served for data analysis.
- Implant Survival/Success/Failure rate: Survival was defined as the prosthesis remaining functional without replacement. Prostheses that needed to be refabricated for any reason were considered as failures. Implants with signs of peri-implantitis were considered as failures and were accounted for as unsuccessful implants using several additional success criteria described by Buser, et al.[41] in previous studies (absence of persistent complaints/absence of peri-implant infection/absence of mobility/absence of continuous radiolucency and pronounced peri-implant marginal bone reduction > 2 mm around the implants).

### Statistical analyses

All parameters were recorded in a descriptive statistical manner, tabulated, and evaluated. Descriptive statistics were used to present data on complications and failures for maxillary and mandibular implant-supported fixed full-arch prostheses (ISFP). The independent two-sample t-test was used to compare normally distributed continuous variables between Zrm-ISFP and r-ISFP; in the case of non-normality (verification with the Kolmogorov–Smirnov test with Lilliefors correction, a p-value < 10% was used as indicator for non-normal distribution) the exact Mann–Whitney U test was used. For comparing categorical variables, Fisher’s exact test was used. For comparing mandible and maxilla within the same group the exact McNemar test was used.

For analyzing prosthesis-related technical complications the statistical unit was the patient, and for analyzing peri-implant marginal bone loss the implants served as the statistical unit. The type I error was set to 5% (two-sided) without adjustment for multiple testing. For statistical analysis, the statistical computing software R Version 4.4.2 (R Foundation for Statistical Computing, Vienna, Austria, URL http://www.R-project.org) was used.

## Results

### Patient follow-up/drop-outs

Out of 18 patients of the test group (TG, Zrm-ISFP) initially included in the follow-up program 4 patients dropped out after 1 or 3 years of loading. Subsequently the TG consisted of 14 patients with bimaxillary metal resin full-arch implant supported prostheses incorporating zirconium molars/quadrant fulfilling a continuous retrospective 5-year follow-up. For the control group (r-ISFP) 5-year data of 15 comparable patients were retrospectively collected from the patient charts allowing for group comparison.

**Fig. 2:**
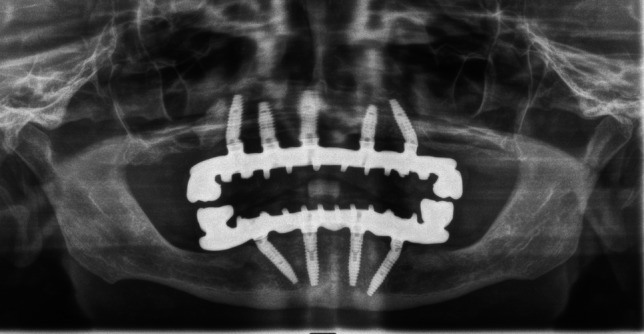
Orthopantomography of bimaxillary ISFP including zirkonium molars (Zr-ISFP)

**Fig. 3:**
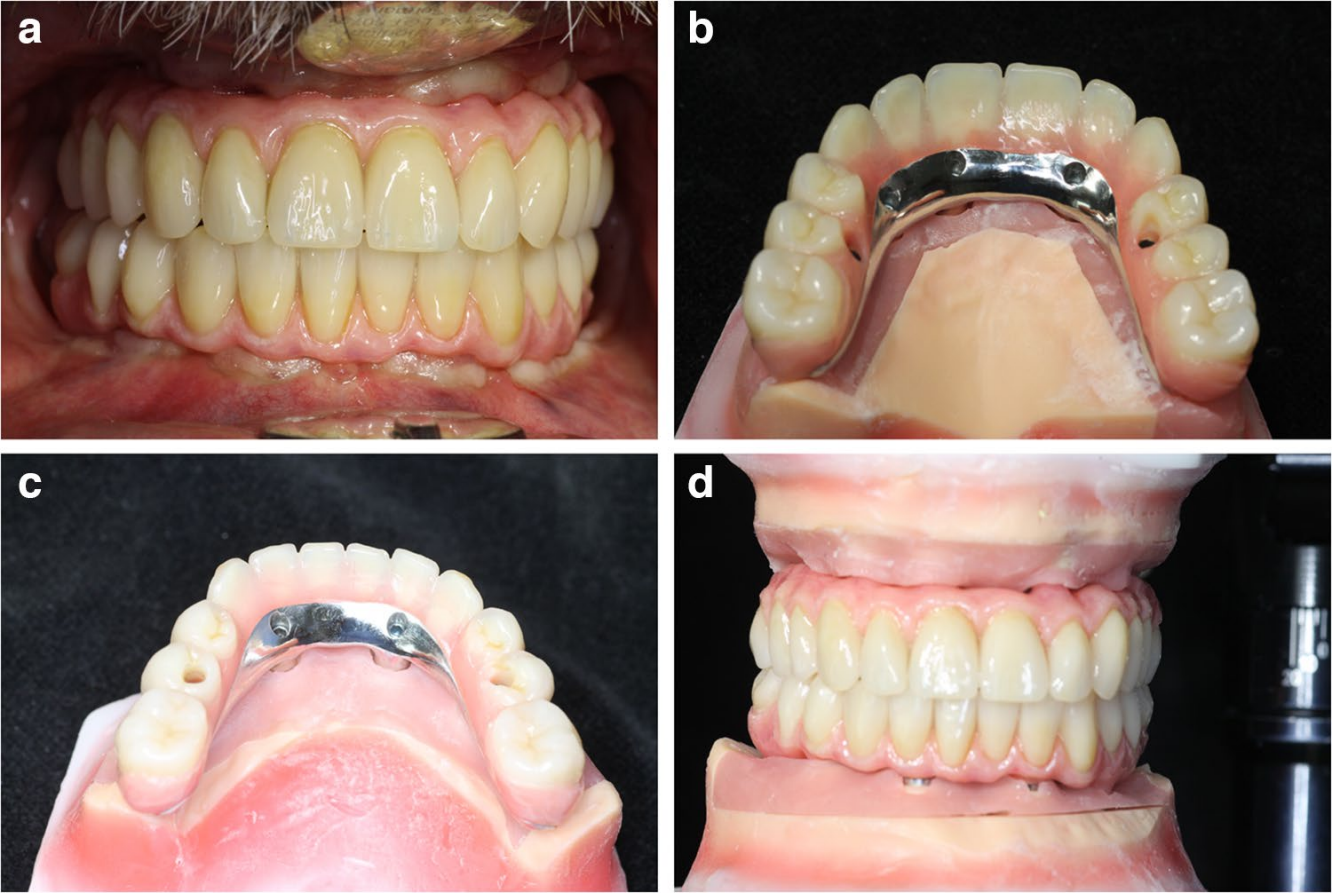
**a**-**d** Clinical situations of bimaxillary ISFP with zirconium molars (Zrm-ISFP)

In total, charts of 29 patients with bimaxillary ISFP subdivided into 14 patients of the TG and 15 patients of the CG were retrospectively evaluated. For TG and CG, in total 116 implants in the mandible (29 x “All-on-4”: 14 × TG-Zrm-ISFP; 15 × CG r-ISFP) and 143 implants in the maxilla (“All-on 4/5/6”: TG: 69 implants/CG: 74 implants) were clinically and radiographically followed for 5 years. Table 3 shows the detailed distribution as characteristics of implants (implant length/diameter) placed in the edentulous maxilla/mandible available for follow-up. There was no difference between the number, distribution as well as length and diameter of implants placed between TG (Zrm -ISFP) and CG (r-ISFP). All implants (n = 259) and prostheses (n = 58) in 29 patients followed survived the 5-year period and provided for a survival rate of 100% both for the TG (n = 14) and the CG (n = 15) and fulfilled the prerequisite for study inclusion. Figure 2 presents orthopantomography of bimaxillary ISFP with zirconium molar in each quadrant. Figure 3a-d present the clinical situations of metal-resin ISFP with zirconium crowns incorporated in the molar region/quadrant.

**Table 3:** Distribution and characteristics of Implants placed for bimaxillary ISFP with (Zrm-ISFP) or without ceramic molar (r-ISFP)

Mandible:	Zrm—ISFP	r—ISFP
“All-on 4”	n = 14	n = 15
Implants: (n):	56	60
Diameter(mm)/length(mm):		
3.8 mm/13 mm:	12 (21.4%)	8 (13.3%)
3.8 mm/16 mm:	28 (50%)	32 (53.3%)
4.3 mm/13 mm:	8 (14.3%)	12 (20%)
3.3 mm/16 mm:	8 (14.3%)	8 (13.3%)
Maxilla:	Zrm—ISFP	r—ISFP
“All on 4/5/6”	n = 4/7/3	n = 4/8/3
Implants:	n = 69	n = 74
Diameter(mm)/length(mm):		
3.8 mm/11 mm:	8 (11.6%)	8 (10.8%)
3.8 mm/13 mm:	28 (40.6%)	36 (48.6%)
3.8 mm/16 mm:	11 (15.9%)	8 (10.8%)
4.3 mm/11 mm:	4 (5.8%)	2 (2.7%)
4.3 mm/13 mm:	12 (17.4%)	16 (21.6%)
4.3 mm/16 mm:	6 (8.7%)	4 (5.4%)

### Primary outcome measures

Table 4 shows the prevalence and the kind of prosthesis-related technical complications (PRTC) of ISFP with zirconium molar (Zrm-ISFP) and with acrylic (r-ISFP) prostheses.

**Table 4:** Implant- and prosthodontic- related technical complications for metal-resin prostheses either with (TG:Zrm-ISFP) or without zirconium molars (CG:r-ISFP) at 1-year,1–3-year and 3–5- year follow- up evaluations

	−1 yr				> 1–3 yr			> 3–5 yr				Total			
	CG			TG	CG		TG	CG			TG	CG			TG
Patients	n = 15			n = 14	n = 15		n = 14	n = 15			n = 14				
Implant component maintenance												Total			
Implant fracture	0		0	> 0.999	0	0	> 0.999	0	0		> 0.999	0	0		> 0.999
Abutment loosening	0		0	> 0.999	1	0	> 0.999	1	1		> 0.999	2	1		> 0.999
Denture screw loosening	4		6	0.731	6	4	0.732	9	5		0.363	19	15		0.571
Abutment fracture	0		0	> 0.999	0	0	> 0.999	0	0		> 0.999	0	0		> 0.999
Total	4		6	0.732	7	4	0.529	10	6		0.435	21	16		0.504
Compl./Prostehses/time:	0.27		0.40		0.23	0.14		0.33	0.21			0.28	0.22		
Effect over time:	CG:p > > 0.999		TG:p = 0.037												
Implant prosthodontic maintenance															
Denture framework fracture	0	0		> 0.999	0	0	> 0.999	0		0	> 0.999	0		0	> 0.999
Resin tooth fracture	6	0		0.024	15	1	< 0.001	24		3	< 0.001	45		4	< 0.001
Resin Teeth repair in office	3	2		> 0.999	8	2	0.081	10		4	0.127	21		8	0.015
Total	9	2		0.042	23	3	< 0.001	34		7	< 0.001	66		12	< 0.001
Compl./Prostheses/time	0.6	0.13			0.77	0.11		1.13		0.25		0.88		0.17	
Effect over time:	CG:p = 0.008	TG:p = 0.678													
Denture base fracture	0	0		> 0.999	0	0	> 0.999	0		0	> 0.999	0		0	> 0.999
Denture rebasing/reduction	8	12		0.412	15	11	0.441	4		7	0.325	27		30	0.522
Denture cleaning	2	0		0.492	10	14	0.286	8		5	0.534	20		19	> 0.999
Screw holes repair	0	2		0.492	3	7	0.173	19		17	> 0.999	22		26	0.403
Total	10	14		0.430	28	32	0.365	31		29	> 0.999	69		75	0.080
Compl./Prostheses/time:	0.67	0.93			0.93	1.14		1.03		1.04		0.92		1.07	
Effect over time:	CG:p = 0.075	TG:p > 0.999													
Total:												135		87	0.0002

In total, the prosthodontic-related technical complications such as resin tooth fracture (45 vs. 4) as well as resin teeth repair in the office (21 vs. 8) differed significantly (p < 0.001) between Zrm-ISFP and r-ISFP over the 5-year follow-up period. For the r-ISFP (CG) an ongoing increase of the prevalence of prosthesis-related technical complications such as tooth fracture over the 5-year observation period was noticed (Tab4). In detail, resin tooth fractures increased from 6 events in the early period (−1 yr) up to 15 events in the mid-term period (> 1–3 yrs) and to 24 events in the late period (> 3–5 years). In total, an evidently higher time-related ongoing increase (−1 yr: n = 9; > 1-3 yrs: n = 23; > 3-5 yrs: n = 34) of the prevalence of PRTC was noted in the CG compared to the TG (−1 yr: n = 2; > 1-3 yrs: n = 3; > 3-5 yrs: n = 7) representing a significant effect over time for the CG (r.ISFP; p = 0.008) than for the TG (Zrm-ISFP; p = 0.678).

However, prosthodontic-related complications non-influenced by occlusal design such as denture rebasing/reduction (30 vs 27), or the need of denture cleaning as a result of resin discoloration (19 vs. 20) and screw hole repair (26 vs. 22) did not show different prevalence between Zrm-ISFP (TG) and r-ISFP (CG;Tab4) even without a significant effect over time (CG: p = 0.075,TG:p > 0.999, Tab4).

The number of patients (TG [Zrm-ISFP]: n = 9, CG [r-ISFP]: n = 10) involved in resin tooth fracture/tooth repair in r-ISFP (n = 66) or Zrm-ISFP (n = 12) did not differ between the two groups (Zrm-ISFP: 9/14 [63%]; r-ISFP:10/15 [66.6%]). Consequently, the prevalence of patients with “prostheses free of technical complications “ was 37% (Zrm-ISFP) and 33.4% (r-ISFP), respectively. In the TG [Zrm-ISFP) 8/9 (88.9%) patients had only one event with one fracture/repair and one patient (11%) required postinsertion maintenance on two occasions with 4 tooth repairs/fractures. In contrast, the total number of tooth fractures/repairs (n = 66) in the r-ISFP (CG) of 15 patients was restricted to 10 patients and could be distributed as follows: 4/10 (40%) patients with 1 event showed 14 teeth fractures/repairs; 4/10 (40%) patients presented with 2 events with 18 fractures/repairs and 2/10 patients (20%) presented 3 and 4 events with 16 and 18 fractures/repairs.

Although the prevalence of patients needing maintenance/repair did not differ between both groups (Zr-ISFP:37%, r-ISFP:33.4%), there were individually more frequent complications in r-ISFP than in Zr-ISFP. A significantly (p < 0.01) lower prevalence of single complication events (8/9 [88.9%] vs 0.4/10 [40%]) as well as recurrent prosthetic technical complications (1/9 [11%] vs. 6/10 [60%],r-ISFP) was found for Zr-ISFP than for r-ISFP.

### Secondary outcome measures

#### Implant-related complications

There were no implant and/or abutment fractures. Implant component maintenance such as abutment/denture screw loosening did not differ (p > 0.999; p = 0.571) between prostheses with (Zrm-ISFP) or without ceramic molar (r-ISFP) incorporation in ISFP.

#### Peri-implant marginal bone level(mm)

Peri-implant marginal bone level reductions of maxillary and mandibular implants supporting Zrm-ISFP and r-ISFP are presented in Table 5. The radiographically measured peri-implant marginal bone reduction did not differ between Zrm-ISFP versus r-ISFP at the 1-, 3- and 5-year evaluation both for maxillary and mandibular implants. In addition, the marginal bone level reduction also did not differ over the observation periods (1-yr vs. 3-yr vs. 5-yr) in r-ISFP and Zrm-ISFP. Interestingly, no significant difference was found between Zrm-ISFP and r- ISFP with regard to the MBL for different (two) implant designs used. The MBL of implants used in in the early phase of this study for CG (before 2017) with smooth collar of 0.4 mm (Camlog, Promote + ® for r-ISFP, n = 134) did not differ from the implant design used in the later time period for the TG (Camlog progressive line® without smooth collar, time after 2017;n = 125).

**Table 5:** Peri-implant marginal bone reduction (MBL) for bimaxillary (maxillary and mandibular) ISFP at 1-, 3- and 5- year post insertion follow-up (FUP)

Mandible:	Zrm-ISFP (n = 14)	r-ISFP (n = 15)	Total (n = 29)
Implants:	n = 56	n = 60	n = 116
MBL:	Mean/SD:mm (range)	Mean/SD:mm (range)	Mean/SD:mm (range)
FUP: 1a:	0.65 ± 0.20 (0.3–1.0)	0.76 ± 0.32 (0.3–1.8)	0.70 ± 0.27 (0.3–1.8)
3a:	0.69 ± 0.21 (0.3–1.0)	0.82 ± 0.41 (0.3–2.5)	0.76 ± 0.33 (0.3–2.5)
5a:	0.71 ± 0.21 (0.3–1.2)	0.87 ± 0.45 (0.3–2.5)	0.80 ± 0.37 {0.3–2.5)
Maxillae:	Zrm-ISFP (n = 14)	r-ISFP (n = 15)	Total (n = 29)
Implants:	n = 69	n = 74	n = 143
MBL:	Mean/SD:mm (range)	Mean/SD:mm (range)	Mean/SD:mm (range)
FUP:1a:	0.57 ± 0.19 (0.0–1.0)	0.45 ± 0.22 (0.0–1.2)	0.51 ± 0.21 (0.0–1.2)
3a:	0.61 ± 0.16 (0.1–1.0)	0.51 ± 0.20 (0.1–1.2)	0.56 ± 0.19 (0.1–1.2)
5a:	0.63 ± 0.17 (0.1–1.0)	0.54 ± 0.20 (0.1–1.2)	0.58 ± 0.19 (0.1–1.2)

Only 4 implants (2 patients) in each group (Zr-ISFP;TG 1x/r-ISFP CG 1x) presented bone loss > 2 mm indicating signs of peri-implantitis. All implants with signs of peri-implantitis were located in anterior mandibular regions.

## Discussion

The findings of the present study indicate that in bimaxillary metal-resin ISFP the incorporation of a ceramic molar per quadrant significantly reduced the prevalence and formation of prosthetic-related technical complications. However, although the prevalence of patients without technical complications did not differ between ISFP with (37%) and without (33.4%) ceramic molars and these were also within previously reported ranges, the number of prosthetic-related technical complications found for the individual patients was significantly reduced for Zr-ISFP. Preservation of posterior occlusal stability by abrasion-resistant molar crowns may be considered as reason for the reduction of denture acrylic teeth fracture/repair resulting in the rejection of the initial hypothesis that no difference would be observed between ISFP with and without zirconium molar incorporation.

Although a number of studies have reported on the use, the success and the complications of monomaxillary ISFP, only few reports are available on the application of bimaxillary ISFP.^27,28,29^ In separate studies by Malo et al.[27] and Papaspyriakos et al.[28] the clinical outcome and the biological and technical complications were described in detail for 55 and 19 patients with bimaxillary ISFP, respectively. In their retrospective analysis, limited by including inhomogeneous prosthesis materials, significantly more frequent incidences of prosthetic-related mechanical complications were reported with bimaxillary ISFP than with monomaxillary restorations. These findings could also be confirmed by the results of Riemann et al.[29] who also reported a significantly higher complication rate (59%) for patients with bimaxillary ISFP than for patients with monomaxillary restorations (41%). As a consequence of these findings it can be assumed that stability and design of the opposite jaw restoration may affect the prevalence of complications of ISFP which is in accordance with previous findings reported by Davis et al. [25], Chochlidakis et al.[26] and Karre et al. [42].

Based on the findings of Papaspyriakos et al.[28] the increased prosthetic-related technical complication rate with bimaxillary ISFP might be attributed to the lacking proprioceptivity of edentulism on the one hand and to the increased bite force generated by dental implants on the other hand [43–45]. This could be confirmed by comparing the mechanosensation and bite force between patients receiving double full-arch ISFPs and those with complete dentures presenting a significantly higher chewing efficiency and maximum bite force for patients with double full-arch IFCDPs compared with edentulous patients with complete dentures [46].

Nevertheless, according to data of numerous studies, reviews and meta-analyses available the easy and simple fabrication, the good reparability, but especially the acceptable costs may be responsible for the fact that the metal-resin prosthesis still continues to be the most commonly used ISFP prosthesis type [3, 5, 8, 13, 15]. Because high implant survival/success rates have already been reported in several previous studies detailed assessments of complications, subdivided into implant-technical, prosthetic technical and implant-biological complications, have increasingly gained in importance for the metal-resin ISFP [6, 9, 10, 12]. In this context, frequencies of 30–40% for prosthetic technical complications with metal-resin ISFP have been reported in separate studies by Purcell et al.[14], Malo et al.[12], Papaspyriakos et al. [10], Priest et al.[8] as well as Barootschie et al.[22].

However, it is worth mentioning that the use of alternative prosthesis material such as metal-ceramic and veneered ceramic for ISFP fabrication has also been associated with an increased frequency of prosthetic-related technical complication such as ceramic chipping [47, 48]. With respect to this topic the studies of Larsson et al.[17], Malo et al.[12] and Papaspyriakos et al.[10], Mendez Caramez et al.[47] and Pieralli et al. [46] describe a frequency of chipping of up to 30–60% for ceramic veneered zirconium frameworks and metal ceramic frameworks. Consequently, the findings of such a high incidence of ceramic chippings for metal-ceramic and veneered zirconium prostheses have increasingly affected the form of ISFP fabrication [10, 12, 47, 48]. Therefore, when taking all these findings into account, ISFP manufacture is preferentially focused on either metal-resin, which can be repaired more easily and more quickly, or increasingly also on stable monolithic ceramic material by means of CAD/CAM procedures [22–24, 49–51].

However, as shown by the present results, pure metal-resin bimaxillary ISFP is also associated with a significant time- and loading- related increase of occlusal prosthetic technical complications. These findings are consistent with previous results of Purcell et al.[14], who reported that for metal-resin ISFP a significantly increased aftercare was required after 5 years of use than after a time of 2 years. This may be attributed to the fact that with increasing time of use ISFPs with a purely acrylic surface in the posterior areas will show increasing occlusal wear. In further course the posterior wear will lead to the development of increased and potentiated occlusal anterior contacts and consequently to the development of complications such as tooth fractures [14–16]. In this context, an appropriate differentiation of the origin has to be made when discussing the development of complications over a certain observation period. Thus, for complications developing early the higher bite force and the missing proprioceptivity may be responsible for teeth fracture/repair while complications occurring during later phases may be due to increasing wear and loss of occlusal support [13, 14, 26, 43, 44].

Interestingly, however, the findings of prosthodontic technical complications with pure resin ISFP were in obvious contrast to the current findings for the metal-resin ISFP incorporating a zirconium molar per quadrant. Although the prevalence of patients involved in prosthetic-technical complications did not differ between r-ISFP and Zr-ISFP, a significantly lower frequency of acrylic teeth fracture/repair and teeth renewing over time/loading for Zr-ISFP with the zirconium crown was seen based on an individual patient level. Preservation of the posterior occlusal stability induced by zirconium crowns may be responsible for the lower rate of recurrence and the reduced number of prosthodontic related occlusal complications.

However, a full-arch monolithic zirconium prosthesis with stable, abrasion-resistant occlusion may also be fabricated and used as a valid prosthetic alternative for ISFP [17–19]. For the full-arch zirconium ISFP high implant-prosthetic success rates with a low incidence of technical complications and high patient satisfaction have been reported indicating the advantages of the zirconium ISFP versus the traditional metal-resin prosthesis [11, 18, 19, 22]. However the fabrication of complete mono- and/or bimaxillary zirconium ISFP may frequently be limited by anatomical, technical and – primarily—financial aspects [22, 52, 53]. Additional disadvantages of monolithic zirconium prostheses may also involve the impossibility of correcting errors of the passive framework fit and inadequate denture design influencing and affecting both aesthetic outcome and phonetic situations. In this context, the production of a CAD/CAM acrylic prostheses prototype has been recommended for avoiding new denture fabrication; however, this will subsequently increase the fabrication costs again [52, 53].

In general, it should be mentioned that a metal framework construction will allow for delicate, stable, patient-friendly and more cost-efficient fabrication of conventional metal-resin prostheses [3, 5, 22, 23, 53, 54]. With regard to the results of the present study, the combination of a metal framework and the incorporation of only one zirconium molar per quadrant into the resin prosthesis may allow for the manufacture of a „hybrid type “ featuring the advantages and benefits of both metal-resin and zirconium prostheses. Apart from the stability and the delicate and defined framework design with the metal framework, the incorporation of a singular ceramic molar will provide for an occlusal stability showing clearly favorable effects and providing for a significant reduction of the occlusion-related prosthodontic complication rate [30, 31, 55–57]. Hence, the fact that a stable occlusion surface in the first molar area will reduce the frequently encountered prosthetic complications of occlusal wear already described in the studies of AlHelal et al.[30] and AlBader et al.[31] can be confirmed. In both studies modification of ISFP provided the molar with a metal crown design incorporated in a metal resin prosthesis [30, 31].In the present study, however, the prosthesis metal framework was ideally fabricated and designed in a manner that an additional incorporation of a zirconium crown was allowed. In contrast to the metal crowns of AlHalil et al.[30] and AlBader et al.[31], the incorporation of a ceramic crown in the molar area not only fulfilled the functional requirements regarding occlusal stability, but also the aesthetic demands of the patients and the clinicians.

However, the present results also show that the prevalence of prosthetic technical complications not being associated with the modified prosthetic occlusal surface, such as rebasing and denture cleaning, showed no significant difference between metal-resin prostheses with and without ceramic molars [3, 5]. The initial high incidence of denture rebasing found for both modalities of denture design evaluated and included may be attributed to the immediate implant placement procedures followed by an ongoing crestal bone resorption. Moreover, the present results also showed that the different occlusal design had no influence on the implant loss/fracture rate and on the marginal peri-implant bone loss [22, 23, 38, 52]. The absence of any difference for implant loss/fracture rate and for the marginal peri-implant bone loss between ISFP with and without zirconium molar is in accordance with the comparative evaluations of implant success and peri-implant findings between pure metal-resin and full zirconium ISFP presented by Barootschi et al. [22] and Bengeni et al.[24].

The modified metal resin prosthesis used for bimaxillary full-arch fixed implant-supported dentures seems promising, combining beneficial effects as hybrid prosthesis evident for metal-resin and complete zirconium prostheses. In particular, the modified prosthesis reduces the development of typical implant-prosthetic technical complications due to the stabilization of the occlusal surface by means of a ceramic tooth and as a result of its metal-resin properties offers a predominantly cost-effective, easily manufactured and aesthetically acceptable ISFP variant. Major advantages may include that the metal-resin properties of the ISFP will allow simple repairs and will especially provide the possibility of basal underlining.

As limiting factors some points have to be considered: 1) in the present study only self-admitted bruxers were excluded without knowledge of the gray area for this topic in edentulism and – 2) no detailed allocation of teeth (anterior vs. posterior, maxilla vs. mandible) in the need of repair was evaluated and −3) the retrospective study design without a randomization protocol. Therefore, additional studies with higher case numbers, randomized prospective study design and prolonged follow-up times will be required to confirm the results obtained.

## Conclusion

The modified metal-resin implant supported fixed prostheses incorporating zirconium molars/quadrants used for bimaxillary ISFP reduce the prevalence of prosthetic related technical complications. This type of modified prosthesis combines beneficial effects such as cost effectiveness and reparability of metal-resin and occlusal stability and reduced wear of complete zirconium prostheses.

## Data Availability

No datasets were generated or analysed during the current study.
