# Correction to: Clinical outcome and technical complications of bimaxillary full‑arch implant‑supported metal‑resin fixed dental prostheses with or without ceramic molars: 5‑year results

**DOI:** 10.1007/s00784-025-06448-5

**Published:** 2025-06-27

**Authors:** Stefan Krennmair, Michael Weinländer, Lukas Postl, Michael Malek, Thomas Forstner, Helfried Hulla, Gerald Krennmair

**Affiliations:** 1 Department of Oral and Maxillofacial Surgery, Keplerklinikum Linz, Johannes Kepler University (JKU) Linz, Linz, Austria; 2 NumBiolab Research Associate, Ludwig-Maximilian University (LMU), Munich, Germany; 3 Dental School, Medical University Graz, Graz, Austria; 4 Head of Department of Oral and Maxillofacial Surgery, Keplerklinikum Linz, Johannes Kepler University (JKU) Linz, Linz, Austria; 5 Department of Applied Systems and Statistics, Johannes Kepler University Linz (JKU), Linz, Austria; 6 Head of Department of Prosthodontics, Dental School, Sigmund Freud Medical University of Vienna, Vienna, Austria

**Correction to: Clin Oral Invest**

10.1007/s00784-025-06409-y

The author Stefan Krennmair was mistakenly replaced by Gerald Krennmair, leading to both names appearing in the published paper.

The original article has been corrected.

